# Exploring the Integrated Role of AKT2, CD44v6, And MT1-MMP as Predictors of Axillary Lymph Node Metastasis in Invasive Breast Carcinoma of No Special Type

**DOI:** 10.30699/IJP.2022.551244.2866

**Published:** 2022-10-05

**Authors:** Primariadewi Rustamadji, Elvan Wiyarta, Kristina Anna Bethania

**Affiliations:** 1 *Department of Anatomic Pathology, Faculty of Medicine Universitas Indonesia, Dr. Cipto Mangunkusumo National Hospital, Jakarta, Indonesia*; 2 *Department of Medical Science, Faculty of Medicine Universitas Indonesia, Dr. Cipto Mangunkusumo National Hospital, Jakarta, Indonesia*

**Keywords:** AKT2, Breast cancer, CD44v6, Immunohistochemistry, Lymph-node, Metastasis, MT1-MMP

## Abstract

**Background & Objective:**

Invasive breast carcinoma of no special type (IBC-NST) is the most common type of breast cancer, which mainly causes axillary lymph-node metastasis (ALNM). Building on our previous research, we wanted to explore the optimal combination of AKT2, CD44v6, and MT1-MMP for the ALNM prediction.

**Methods:**

The presence or absence of ALNM was used to separate 46 paraffin blocks containing IBC-NST primary tumors into two groups. Age, tumor grade, tumor size, receptor status (ER, PR, HER2, Ki-67, TOP2A), and test biomarker expression were evaluated. Biomarker expressions were assessed by IHC staining and categorized according to their respective cut-offs from our previous study, while other data were collected from archives. Data was gathered and analyzed using univariate, multivariate, and AUROC models.

**Results:**

The expression of CD44v6 (OR: 12.77, 95% CI: 2.18-87.12, *P*=0.005) was identified as the independent variable for ALNM. Meanwhile, AKT2 expression (OR: 3.22, 95% CI: 0.36-22.41, *P*=0.237) and MT1-MMP expression (OR: 5.35, 95% CI: 0.83-34.54, *P*=0.078) did not demonstrate a statistically significant independent association in respect to ALNM. Combining AKT2 and MT1-MMP on CD44v6 increased overall accuracy by 4% compared to CD44v6 alone (AUROC 0.89 vs. 0.85).

**Conclusion:**

The combined usage of AKT2, CD44v6, and MT1-MMP revealed no significant change compared to CD44v6 alone. Due to the cost and practicality, we propose using CD44v6 as a predictor biomarker of ALNM in IBC-NST.

## Introduction

Breast cancer is the most prevalent malignancy in women and the leading cause of cancer death. Global Burden Cancer (GLOBOCAN) data for both sexes combined in 2020 shows that female breast cancer is the most often diagnosed cancer, accounting for 11.7% of total cases and 6.9% connected to mortality (1). Breast cancer is a tumor of the breast tissue, classified into two types: invasive and non-invasive breast carcinoma. Invasive breast carcinoma is a more frequent type of breast cancer that consists of several subtypes. Among the invasive breast carcinoma subtypes, 80% are invasive breast carcinoma of no special type (IBC-NST) (2).

The worse the grade of IBC-NST is, the worse the patient’s prognosis is (3, 4). This is due to the increased likelihood of metastasis in IBC-NST. Metastasis is a stage of cancer progression in which cancer cells move and grow in other tissues (5). Metastasis, which takes place when new cancer cell colonies form in a other organs, can affect the patient’s prognosis (6). The most prevalent organ for metastasis in IBC-NST is the axillary lymph node, called axillary lymph node metastasis (ALNM) (7).

Several non-invasive methods have been used to predict ALNM in IBC-NST, including using biomarkers. Our recent work discovered AKT2, CD44v6, and MT1-MMP potentials independently, followed by studying their H-score cut-offs (8). In this study, we would like to explore further integrated role of these three biomarkers in predicting ALNM in IBC-NST.

AKT is a protein found in the cell membrane and cytoplasm that will activate a variety of downstream protein substrates resulting in cancer development (9). However, many investigations have yielded inconclusive data about the function of AKT in breast cancer, which is possible because AKT is said to have three isoforms: AKT1, AKT2, and AKT3, each of which has distinct and even conflicting functions (10, 11). Of the three isoforms, only AKT2 was suspected of inducing cell migration and metastasis through the induction of vimentin and F-actin (11). Its role conflicts with other isoforms, such as AKT1, which plays a role in cancer cell proliferation but does not affect metastasis, and AKT3, which plays a more significant part in the progression of triple-negative breast cancer (11). 

CD44 is a transmembrane glycoprotein receptor family that binds to hyaluronic acid, resulting in intracellular signalling connected to various biological activities such as cell adhesion, migration, and invasion (12). CD44 consists of 2 isoforms after undergoing alternative splicing, namely standard isoforms (CD44s) and several variant isoforms (CD44v) (13). It has been proposed that upregulation of one or more CD44v has a role in developing certain cancers (14). There are numerous exon variations in CD44v, but the CD44 having variant exon 6 (CD44v6) has been studied extensively (14). CD44v6 has been found to mediate pancreatic and colorectal cancer metastasis (14). However, the involvement of CD44v6 in breast cancer ALNM has not been widely explored.

MMP is a zinc-containing proteolytic enzyme family responsible for damaging or breaking down extracellular matrix components (15). MT1-MMP is a proteolytic enzyme responsible for damaging or breaking down the components of the extracellular matrix, such as laminin and fibronectin, which certainly make it easier for tumoral cells to invade and eventually metastasize to other organs (16). 

This study explores the optimal combination for the ALNM prediction and validates the cut-off H-score for each biomarker. We hypothesized that the integrated use of AKT2, CD44v6, and MT1-MMP could predict ALNM in IBC-NST. AKT2, CD44v6, and MT1-MMP expressions, as assessed by immunohistochemistry (IHC) staining, were quantified using H-score. We utilize the H-score cut-off from our prior study to separate data regarding AKT2 expression (8), CD44v6 (17), and MT1-MMP. This study is expected to clarify further and validate the integrated role of AKT2, CD44v6, and MT1-MMP in predicting ALNM in IBC-NST.

## Material and Methods

Study Design and Data Collection

This cross-sectional study was conducted in the Anatomical Pathology Laboratory, Faculty of Medicine, Universitas Indonesia, from June 2020 to June 2021. The Ethics Committee approved the experimental protocols of the Faculty of Medicine, Universitas Indonesia, with protocol number 20-09-1169 in June 2020. The study was undertaken with the understanding and written consent of each subject. The study conforms to The Code of Ethics of the World Medical Association (Declaration of Helsinki) (18). The data retrieved included patient’s age, tumor subtype, tumor grade, tumor size, estrogen receptor (ER) status, progesterone receptor (PR) status, human epidermal growth factor receptor 2 (HER2) status, Ki67 status, topoisomerase II alpha (TOP2A) status, and ALNM. In addition, AKT2, CD44v6, and MT1-MMP expression data were obtained by quantifying the IHC staining results.

Samples

Primary tumor paraffin blocks from female breast mastectomy patients who were histopathologically diagnosed as IBC-NST for the first time, either with or without ALNM, were used in this investigation. We exclude samples from individuals who have a different histological state than IBC-NST (e.g., papillary carcinoma, medullary carcinoma, invasive lobular carcinoma, etc.), systemic comorbidities (diabetes, hypertension, etc.), or questionable paraffin blocks (e.g., broken or weakened paraffin blocks, etc.).

The samples were classified based on the presence or absence of ALNM in post-operative findings. The sample size was calculated using alpha = 5%, confidence interval = 95%, and power = 80%, and a minimum of 23 samples were collected in each category. In this study, 23 samples from the ALNM group and 23 samples from the non-ALNM group were utilized. To avoid bias, the grouping findings were only accessible to one researcher (E.W.). Other researchers were unaware of which categories each study fell into until the analysis was done.

Slide Preparation and IHC Staining

The staining technique is followed by Primariadewi* et al.* (2021) and Kusmardi* et al.* (2021) (8, 19-22). According to typical protocols, a paraffin slice of a breast cancer specimen was deparaffinized in xylol (Merck, Jakarta, Indonesia) and rehydrated in 96%, 70% absolute alcohol series (Merck, Jakarta, Indonesia) and distilled water for 5 minutes. Heat-induced antigen retrieval was carried out in a 96°C Decloaking Chamber for 20 minutes in Tris EDTA (Brataco Inc., Jakarta, Indonesia) pH 9.0. Following antigen retrieval, sections were treated for 15 minutes with peroxidase block before being washed with phosphate-buffered saline (PBS) pH 7.4. The slide was incubated for 1 hour with anti-AKT2 antibody (ab175354, Abcam, Cambridge, UK), anti-CD44v6 antibody (ab30436, Abcam, Cambridge, UK), and anti-MT1-MMP (ab51074, Abcam, Cambridge, UK), followed by post-primary and Novolink polymer incubation. Diaminobenzidine (Abcam, Jakarta, Indonesia) was used as the chromogen responsible for the brown color, and tissue sections were counterstained with hematoxylin (Abcam, Jakarta, Indonesia) and bluing with 5% Lithium carbonate (Merck, Jakarta, Indonesia) before being examined under a microscope.

Quantification of AKT2, CD44v6, and MT1-MMP Expression

Two experienced researchers (P.R. and K.A.B.) assessed the IHC staining. Each preparation was examined under a light microscope at a total magnification of 400x and documented using a computer running Leica LAZ EZ software and a camera with a white balance format combined with a Leica DM750 microscope. AKT2, CD44v6, and MT1-MMP expression were measured randomly in at least 500 tumoral cells from five separate visual fields (400x). A minimum of 100 tumoral cells were used to represent each area. Brown staining of the tumoral cell membrane and cytoplasm indicated the presence of AKT2, CD44v6, and MT1-MMP expression (23). Based on the strength of the brown color measured in each field of view using ImageJ’s cell counter, staining intensity was classified as no staining (0), low positive (1+), positive (2+), and high positive (3+) (24). The H-score was used to measure the expression of AKT2, CD44v6, and MT1-MMP. The H-score is computed using the following formula: H-score = (percentage of low positives x 1) + (percentage of positives x 2) + (percentage of high positive x 3) (25). The H-scores of the full sample were computed separately by two observers (P.R. and K.A.B.). To minimize bias, the findings of previously examined computations were gathered and distributed to other researchers (E.W.) until the full sample was evaluated. For future analysis, the mean H-score of the two observers will be utilized. 

Statistical Analysis

Before analysis, data collection was entered into a primary table using Microsoft Excel (Microsoft Corp, Redmond, WA, USA). The tabulated data were analyzed and visualized using the Statistical Package for Social Sciences / SPSS version 20 (IBM Corp, Armonk, NY, USA). To test the data’s reliability, the variability of the H-score between the two observers was compared to the Intraclass Correlation Coefficient (ICC). The ICC model employed is a two-way mixed average with absolute agreement. The 95% interval of the ICC estimate was used to categorize ICC values: less than 0.5, between 0.5 and 0.75, between 0.75 and 0.9, and higher than 0.90 indicate low, moderate, suitable, and excellent reliability, respectively (26). We used cut-off H-scores for each of the biomarkers studied in our previous study. The cut-off H-scores for AKT2, CD44v6, and MT1-MMP were 104.62, 133.89, and 202.22, respectively (8). The H-scores from the two observers were averaged and grouped as high or low based on the H-score cut-off. These groups describe the expression of AKT2, CD44v6, and MT1-MMP for each sample. 

≥ of the biomarkers was also analyzed and compared based on overall accuracy, sensitivity, specificity, positive predicted value (PPV), and negative predicted value (NPV) (27). The cut-off of the biomarkers is also calculated based on the highest Youden Index (28) and the lowest K-Index (29). 

## Results

IHC Staining and H-Score Reliability

**Fig. 1:**
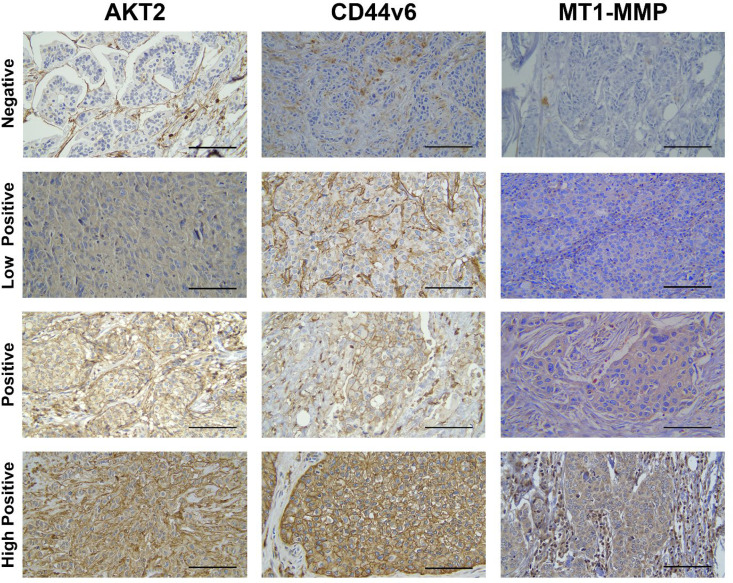
IHC staining for AKT2, CD44v6, and MT1-MMP expression in IBC-NST tumor cells at 400x magnification. Scale bar represents 50 μm for all images

All forty-six samples underwent IHC staining for AKT2, CD44v6, and MT1-MMP expression. Each sample's clinicopathologic characteristics are expressed in Table 1. The results of representative IHC staining can be seen in Figure 1. Each image provides a sample of tumoral cells with different staining groups, including negative, low positive, positive, and high positive. The images represent a collection of visual fields taken from the same slide. This also demonstrates that numerous cells of varying intensity may be recognized on a single slide, and in many cases, even in a single visual field. The intensity of the brown color in these slides is measured and converted into an H-score for future investigation.

Two observers (P.R. and K.A.B.) assessed all forty-six samples independently. For AKT2, excellent reliability was found between the two measurements, with the average measure ICC being 0.987 with a 95% confidence interval from 0.977 to 0.993 (F (45,45) = 161.477, *P*<0.001). For CD44v6, excellent reliability was found between the two measurements, with the average measure ICC being 0.975 with a 95% confidence interval from 0.956 to 0.986 (F (45,45) = 78.006, *P*<0.001). For MT1-MMP, excellent reliability was found between the two measurements, with the average measure ICC being 0.934 with a 95% confidence interval from 0.882 to 0.983 (F (45,45) = 30.518, *P*<0.001).

Association between AKT2, CD44v6, and MT1-MMP Expression with ALNM in IBC-NST

In order to investigate the association between AKT2, CD44v6, and MT1-MMP expression and ALNM, a thorough analysis is needed, which includes identifying, reducing, and controlling confounding factors. In this study, several confounding factors were identified, including age, tumor grade, tumor size, ER status, PR status, HER2 status, Ki67 status, and TOP2A status, as shown in Table 1. Together with the expression of AKT2, CD44v6, and MT1-MMP, the confounding factors were subjected to a univariate test for their influence on ALNM, the results of which are shown in Table 2.

The univariate test findings revealed that none of the confounding factors were significantly associated with ALNM. AKT2 (*P*=0.001), CD44v6 (*P*<0.001), and MT1-MMP (*P*=0.002) expression, on the other hand, exhibited a significant association with ALNM. Multivariate regression analysis was used further to analyze the association between the three integrated biomarkers. According to the study findings, the expression of CD44v6 (OR: 12.77, 95% CI: 2.18-87.12, *P*=0.005) was identified as the independent variable for ALNM. Meanwhile, AKT2 expression (OR: 3.22, 95% CI: 0.36-22.41, *P*=0.237) and MT1-MMP expression (OR: 5.35, 95% CI: 0.83-34.54, *P*=0.078) did not demonstrate a statistically significant independent function in respect to ALNM. The multivariate model’s validity was excellent, as the Hosmer–Lemeshow test (*P*=0.526) revealed that the numbers of ALNM were not substantially different from those predicted by the model and had satisfactory goodness of fit. This model may also predict ALNM by including important factors into the probability calculation for multiple logistic regression (30), as shown in formula 1. The value of X indicates the status of the CD44v6 expression, which is 1 if it is high and 0 if it is low. Based on this calculation, patients with high CD44v6 expression had an 86.36 % chance of having ALNM. Patients with low CD44v6 expression, on the other hand, had a 16.67% chance of having ALNM.


(1) $$P(Y)=\frac{e^{\mathrm{X.}\left(2.62\right)-2.54}}{1+e^{\mathrm{X.}\left(2.62\right)-2.54}}$$

**Table 1:** Clinicopathological features of the study population

Clinicopathological characteristic	N or value	%
Age (years)		
** 50**	25	54.35
**< 50**	21	46.65
**Mean (SD)**	49.98 (1.71)	
**Median (Min-Max)**	50 (29-75)	
		
Tumor Grade		
**Grade I**	4	8.70
**Grade II**	16	34.78
**Grade III**	26	56.52
		
Tumor Size (cm)		
**<2**	2	4.35
**2-5**	30	65.22
**>5**	14	30.43
		
ER status		
**Positive**	39	84.78
**Negative**	7	15.22
		
PR status		
**Positive**	31	67.39
**Negative**	15	32.61
		
HER2 status		
**Positive**	15	32.61
**Negative**	31	67.39
		
Ki67 status		
**Positive**	44	95.65
**Negative**	2	4.35
		
TOP2A		
**Positive**	26	56.52
**Negative**	20	43.48

**Table 2:** Univariate and multivariate analysis on several variables for ALNM

Variables	Category	Lymph-Node Metastasis		Univariate Analysis (n=46)		Multivariate analysis (n=46)		
		Yes (%)	No (%)	P-value	OR(95% CI)	Standardized Coefficient	P-value	OR(95% CI)
Age	y.o.	13 (52.0%)	12 (48.0%)	>0.999	0.84 (0.26-2.86)	-	-	-
	<50 y.o.	10 (47.6%)	11 (52.4%)					
Tumor grade	High	14 (53.8%)	12 (46.2%)	0.776	0.70 (0.22-2.26)	-	-	-
	Low	9 (45.0%)	11 (55.0%)					
Tumor size	>5 cm	6 (35.3%)	11 (64.7%)	0.223	2.57 (0.75-8.97)	-	-	-
	≤5 cm	17 (58.6%)	12 (41.4%)					
ER status	Positive	19 (48.7%)	20 (51.3%)	>0.999	0.71 (0.14-3.61)	-	-	-
	Negative	4 (57.1%)	3 (42.9%)					
PR status	Positive	17 (54.8%)	14 (45.2%)	0.529	1.82 (0.52-6.37)	-	-	-
	Negative	6 (40.0%)	9 (60.0%)					
HER2 status	Positive	7 (46.7%)	8 (53.3%)	>0.999	0.82 (0.24-2.82)	-	-	-
	Negative	16 (51.6%)	15 (48.4%)					
Ki67 status	Positive	22 (50.0%)	22 (50.0%)	>0.999	1.00 (0.06-17.02)	-	-	-
	Negative	1 (50.0%)	1 (50.0%)					
TOP2A status	Positive	15 (57.7%)	11 (42.3%)	0.372	2.05 (0.63-6.69)	-	-	-
	Negative	8 (40.0%)	12 (60.0%)					
AKT2 expression	High	20 (71.4%)	8 (28.6%)	0.001*	12.50 (2.83-55.25)	1.17	0.237	3.22 (0.36-22.41)
	Low	3 (16.7%)	15 (83.3%)					
CD44v6 expression	High	19 (86.4%)	3 (13.6%)	<0.001*	31.67 (6.25-160.54)	2.62	0.005*	12.77 (2.18-87.12)
	Low	4 (16.7%)	20 (83.3%)					
MT1-MMP expression	High	14 (82.4%)	3 (17.6%)	0.002*	10.37 (2.37-45.30)	1.68	0.078	5.35 (0.83-34.54)
	Low	9 (31.0%)	20 (69.0%)					
Constant	-	-	-	-	-	-2.54	0.003*	-

**Predictability of ALNM in IBC-NST by AKT2, CD44v6, and MT1-MMP based on AUROC**

**Fig. 2:**
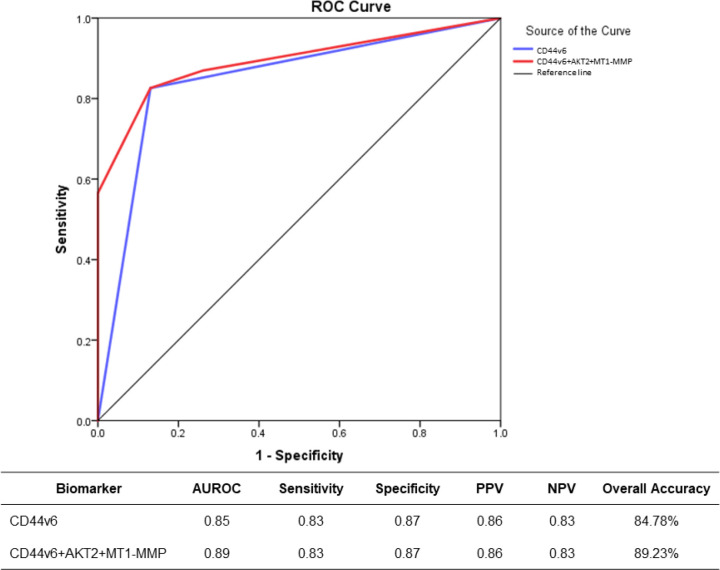
ROC curve to plot the predictability of ALNM by AKT2, CD44v6, and MT1-MMP. Diagonal segments are produced by ties

In this section, we examine the predictability of ALNM by CD44v6 alone compared to that when AKT2 and MT1-MMP are included. Even though multivariate analysis has been performed, the relationship between the test biomarkers and ALNM is examined by the logistic regression association and AUROC analysis. The ability of the test biomarkers to predict ALNM was assessed using nonparametric ROC analysis following National Cancer Institute guidelines (31), as shown in Figure 2. The findings showed that using CD44v6 alone to predict ALNM had an AUROC value of 0.85 (95% CI: 0.73-0.96). On the other hand, using all biomarkers AKT2, CD44v6, and MT1-MMP increased the AUROC score by 4% to 0.89. (95% CI: 0.80-0.99). However, based on the Youden Index and K-Index values, both models have the same cut-off with comparable sensitivity, specificity, PPV, and NPV values.

## Discussion

Using the AKT2 (8), CD44v6, and MT1-MMP cut-offs from our previous study, we attempted to investigate the integration potential of these three biomarkers in prediction ALNM in IBC-NST. We explored the relationship between AKT2, CD44v6, and MT1-MMP using multivariate analysis and the AUROC comparison after identifying and adjusting for confounding factors using univariate analysis. Our findings revealed an intriguing connection between the integration of the three biomarkers and the prediction of ALNM.

Multivariate regression analysis highlighted CD44v6 (OR: 12.77, 95% CI: 2.18-87.12, *P*=0.005) as independent variables in the prediction of ALNM in IBC-NST. AKT2 and MT1-MMP, on the other hand, did not play a significant role in the multivariate analysis for the prediction of ALNM. As a result, the ALNM prediction model calculation (formula 1) only considers CD44v6 status (high or low), as patients with high CD44v6 expression have an 86.36% chance of having ALNM, and patients with low CD44v6 expression have a 16.67% chance of having ALNM. However, the AUROC analysis should also be conducted to analyze the association between these three biomarkers further. This is because sensitivity, specificity, and accuracy in AUROC analysis may provide clinical importance, although multivariate analysis is not statistically significant (32, 33). It was discovered that combining AKT2 and MT1-MMP on CD44v6 increased overall accuracy by 4% compared to CD44v6 alone (AUROC 0.89 vs. 0.85). Nonetheless, this 4% rise has insufficient clinical significance; the two do not have different sensitivity, specificity, PPV, and NPV values. These findings suggest that CD44v6 can predict ALNM independently. The inclusion of AKT2 and MT1-MMP did not improve the predictive performance.

CD44v6 was able to be an independent factor in the prediction of ALNM in IBC-NST as indicated by the significant univariate (*P*<0.001) and multivariate (OR: 12.77, 95% CI: 2.18-87.12, *P*=0.005) analysis. This finding is consistent with the molecular mechanism of CD44v6, which has been shown to improve the capacity of invasive breast cancer cells to proliferate, migrate, invade, and metastasize (34, 35). CD44v6 can activate c-Met, which leads to activating the Ras-SOS signalling cascade, which causes cell proliferation [31] and activates VEGFR-2, promoting metastasis (36). Several translational studies support this finding, including Kaufmann* et al.* (37), who demonstrated that CD44v6 is an independent marker for predicting lymph node metastases in primary breast cancer, and Günthert* et al.* (38), who revealed that transfection of tumor cells with CD44v6 could enhance lymph node metastasis. On the other hand, several studies show different results. Lyzak* et al.* (39) found that CD44v6 was not associated with ALNM. However, this study only looked at breast cancer stages T1a and T1b. After multivariate analysis, Bànkfalvi* et al.* (40) discovered no significant association between CD44v6 and nodal status. However, this might be because CD44v6 was evaluated with other CD44 isoforms (v3, v4, v5, v7, and v9) from the same protein family, potentially suppressing its power. This contrasts with the findings of our investigation, which compared CD44v6 to biomarkers from other families, notably AKT2 and MT1-MMP. Another research that is similar to ours is by Umeda* et al.*(12), who found that CD44v6 expression had no connection with lymph node metastases in IBC-NST patients. Even though we both utilized IHC staining, the quantity of their research samples was half ours, thus affecting their study’s power. Furthermore, not all of their samples came from the axillary lymph node, but some came from the mediastinal lymph node. These works add to the finding of our previous study, which demonstrated the potential of CD44v6 as a biomarker predictor of ALNM in IBC-NST.

AKT2 did not show potential as an independent biomarker (OR: 3.22, 95% CI: 0.36-22.41, *P*=0.237) based on multivariate analysis, although it did have an association with ALNM (*P*=0.001) based on univariate analysis. The relationship between AKT2 and ALNM is contentious, especially compared to the other isoform, such as AKT1 and AKT3. Aside from that, research indicates that AKT2 functions in cell invasion and migration by activating integrin 1-mediated attachment to and invasion via collagen IV (41). AKT2 also promotes metastasis by activating the adhesion-associated 1-integrin and actin-polymerizing LIMK/Cofilin axis (41). This theory is consistent with our previous research, which found that AKT2 expression is an independent variable in the prediction of ALNM (8). However, as compared to CD44v6, AKT2 played a less significant association in this study. The insignificant multivariate analysis result does not necessarily imply that AKT2 plays no role in the prediction of ALNM in IBC-NST. However, it does play a less dominant role when combined with CD44v6. This might be related to differences in CD44v6 and AKT2 expression specificity. In invasive breast carcinoma, CD44v6 is explicitly expressed on luminal epithelial cells (14), whereas AKT2 is present in the epithelial and stromal compartments in a similar ratio (42). This is crucial since most invasive breast carcinomas have a phenotype similar to luminal epithelial cells (43). Furthermore, luminal epithelial proliferation can be seen in highly metastatic cancers (44), making CD44v6 more specific for predicting ALNM than AKT2.

MT1-MMP did not show potential as an independent biomarker (OR: 5.35, 95% CI: 0.83-34.54, *P*=0.078) based on multivariate analysis, although it did have an association with ALNM (*P*=0.002) based on univariate analysis. The association of MT1-MMP with ANLM can be explained by its involvement in activating MMP13 and MMP2, which are involved in extracellular matrix degradation, such as laminin and fibronectin (16). This matrix breakdown will almost definitely make tumor cells more likely to infiltrate and eventually spread to other organs. Our previous study also supports this theory, in which MT1-MMP expression functions as an independent variable in the prediction of ALNM. However, as compared to CD44v6, the role of MT1-MMP in this research was less significant. MT1-MMP’s minor function in biomarker integration, like AKT2, may be related to differences in expression specificity compared to CD44v6. As previously explained, CD44v6 is explicitly expressed on luminal epithelial cells (14), but MT1-MMP, like AKT2, is present in both the epithelium and the stroma (45). This is what affects the function of MT1-MMP in the prediction of ALNM in IBC-NST when combined with CD44v6.

This study has several limitations. Because this research was conducted in a tertiary hospital, the IBC-NST were predominantly high-grade tumors. Further research is needed to cover a more diversified population by analyzing low-grade tumors. Furthermore, even though we have maximized the number of samples feasible in our facility, more extensive sample size research with more diversified biomarkers is still required, revealing the possibility for other biomarkers to be incorporated in the prediction of ALNM on IBC-NST.

## Conclusion

In conclusion, the combined usage of AKT2, CD44v6, and MT1-MMP revealed no significant change compared to CD44v6 alone. Although both can predict ALNM, we propose using CD44v6 as a predictor biomarker of ALNM in IBC-NST due to cost and practicality. More research with a larger population and more diversified biomarkers is required to improve the prediction of ALNM in IBC-NST.

## Conflict of Interest

The authors declared no conflict of interest.

## Funding

The author(s) received no financial support for the research, authorship, and/or publication of this article.

## References

[1] GLOBOCAN Estimated cancer incidence, mortality and prevalence in 2020: International Agency for Research on Cancer-WHO; 2020 [updated 2020/03/.

[2] Sharma GN, Dave R, Sanadya J, Sharma P, Sharma KK (2010). Various types and management of breast cancer: an overview. J Adv Pharm Technol Res.

[3] Oluogun WA, Adedokun KA, Oyenike MA, Adeyeba OA (2019). Histological classification, grading, staging, and prognostic indexing of female breast cancer in an African population: A 10-year retrospective study. Int J Health Sci (Qassim).

[4] Sun Y, Liang F, Cao W, Wang K, He J, Wang H (2014). Prognostic value of poorly differentiated clusters in invasive breast cancer. World J Surg Oncol.

[5] Xu Q, Yuan J-P, Chen Y-Y, Zhang H-Y, Wang L-W, Xiong B (2020). Prognostic Significance of the Tumor-Stromal Ratio in Invasive Breast Cancer and a Proposal of a New Ts-TNM Staging System. Journal of Oncology.

[6] Chiang AC, Massagué J (2008). Molecular Basis of Metastasis. N Engl J Med.

[7] Zhang Y, Li J, Fan Y, Li X, Qiu J, Zhu M (2019). Risk factors for axillary lymph node metastases in clinical stage T1-2N0M0 breast cancer patients. Medicine.

[8] Rustamadji P, Wiyarta E, Bethania KA, Kusmardi K (2021). Potential of AKT2 expression as a predictor of lymph-node metastasis in invasive breast carcinoma of no special type. J Pathol Transl Med.

[9] Song M, Bode AM, Dong Z, Lee M-H (2019). AKT as a Therapeutic Target for Cancer. Cancer Res.

[10] Yang ZY, Di MY, Yuan JQ, Shen WX, Zheng DY, Chen JZ (2015). The prognostic value of phosphorylated Akt in breast cancer: a systematic review. Sci Rep.

[11] Riggio M, Perrone MC, Polo ML, Rodriguez MJ, May M, Abba M (2017). AKT1 and AKT2 isoforms play distinct roles during breast cancer progression through the regulation of specific downstream proteins. Sci Rep.

[12] Umeda T, Ishida M, Murata S, Mori T, Kawai Y, Itoi N (2016). Immunohistochemical analyses of CD44 variant isoforms in invasive micropapillary carcinoma of the breast: comparison with a concurrent conventional invasive carcinoma of no special type component. Breast Cancer.

[13] Chen C, Zhao S, Karnad A, Freeman JW (2018). The biology and role of CD44 in cancer progression: therapeutic implications. Journal of Hematology & Oncology.

[14] Diaz LK, Zhou X, Wright ET, Cristofanilli M, Smith T, Yang Y (2005). CD44 expression is associated with increased survival in node-negative invasive breast carcinoma. Clin Cancer Res.

[15] Ren L, Wang Y, Zhu L, Shen L, Zhang J, Wang J (2018). Optimization of a MT1-MMP-targeting Peptide and Its Application in Near-infrared Fluorescence Tumor Imaging. Scientific Reports.

[16] Jiang WG, Davies G, Martin TA, Parr C, Watkins G, Mason MD (2006). expression of membrane type-1 matrix metalloproteinase, MT1-MMP in human breast cancer and its impact on invasiveness of breast cancer cells. Int J Mol Med.

[17] Rustamadji PA-O, Wiyarta EA-O, Bethania KA-O CD44 Variant Exon 6 Isoform Expression as a Potential Predictor of Lymph Node Metastasis in Invasive Breast Carcinoma of No Special Type.

[18] Rickham PP (1964). Human Experimentation. Code of Ethics of The World Medical Association Declaration of Helsinki. Br Med J.

[19] Kusmardi K, Wiyarta E, Estuningtyas A, Sahar N, Midoen YH, Tedjo A (2021). Potential of Phaleria macrocarpa Leaves Ethanol Extract to Upregulate the Expression of Caspase-3 in Mouse Distal Colon after Dextran Sodium Sulphate Induction. Pharmacognosy Journal.

[20] Kusmardi K, Wiyarta E, Estuningtyas A, Sahar N, Midoen YH, Tedjo A (2021). Potential Inhibition by Phaleria macrocarpa Leaves Ethanol Extract on Ki-67 Expression in Distal Colon Mouse. Pharmacognosy Journal.

[21] Kusmardi K, Wiyarta EA-O, Rusdi NA-O, Maulana AM, Estuningtyas A, Sunaryo H The potential of lunasin extract for the prevention of breast cancer progression by upregulating E-Cadherin and inhibiting ICAM-1.

[22] Rustamadji P, Wiyarta E, Anggreani I (2022). Exploring the Expression of Survivin on Neoadjuvant Chemotherapy in Invasive Breast Carcinoma. Open Access Macedonian Journal of Medical Sciences.

[23] Hashimoto K, Tsuda H, Koizumi F, Shimizu C, Yonemori K, Ando M (2014). Activated PI3K/AKT and MAPK pathways are potential good prognostic markers in node-positive, triple-negative breast cancer. Annals of Oncology.

[24] O'Brien J, Hayder H, Peng C Automated Quantification and Analysis of Cell Counting Procedures Using ImageJ Plugins. JoVE.

[25] Choudhury KR, Yagle KJ, Swanson PE, Krohn KA, Rajendran JG (2010). A Robust Automated Measure of Average Antibody Staining in Immunohistochemistry Images. J Histochem Cytochem.

[26] Koo TK, Li MY (2016). A Guideline of Selecting and Reporting Intraclass Correlation Coefficients for Reliability Research. J Chiropr Med.

[27] Wiyarta E, Kusmardi K, Tedjo A, Sunaryo H (2022). In Vitro and In Vivo Study of Pandanus conoideus Oil Extract in the Maturation of Mouse Peritoneal Macrophages. Open Access Macedonian Journal of Medical Sciences.

[28] Ruopp MD, Perkins NJ, Whitcomb BW, Schisterman EF (2008). Youden Index and Optimal Cut-Point Estimated from Observations Affected by a Lower Limit of Detection. Biom J.

[29] Kallner A, Kallner A (2018). Formulas. Laboratory Statistics (Second Edition).

[30] Peng C-YJ, Lee KL, Ingersoll GM (2002). An Introduction to Logistic Regression Analysis and Reporting. The Journal of Educational Research.

[31] Pepe MS, Feng Z, Janes H, Bossuyt PM, Potter JD (2008). Pivotal evaluation of the accuracy of a biomarker used for classification or prediction: standards for study design. J Natl Cancer Inst.

[32] Seshan VE, Gönen M, Begg CB (2013). Comparing ROC curves derived from regression models. Stat Med.

[33] Zou KH, O'Malley AJ, Mauri L (2007). Receiver-Operating Characteristic Analysis for Evaluating Diagnostic Tests and Predictive Models. Circulation.

[34] Hu S, Cao M, He Y, Zhang G, Liu Y, Du Y (2020). CD44v6 Targeted by miR-193b-5p in the Coding Region Modulates the Migration and Invasion of Breast Cancer Cells. Journal of Cancer.

[35] Louderbough JMV, Schroeder JA (2011). Understanding the Dual Nature of CD44 in Breast Cancer Progression. Molecular Cancer Research.

[36] Martin TA, Harrison G, Mansel RE, Jiang WG (2003). The role of the CD44/ezrin complex in cancer metastasis. Crit Rev Oncol Hematol.

[37] Kaufmann M, Heider KH, Sinn HP, von Minckwitz G, Ponta H, Herrlich P (1995). CD44 variant exon epitopes in primary breast cancer and length of survival. Lancet.

[38] Günthert U, Hofmann M, Rudy W, Reber S, Zöller M, Haussmann I (1991). A new variant of glycoprotein CD44 confers metastatic potential to rat carcinoma cells. Cell.

[39] Lyzak JS, Yaremko ML, Recant W, Baunoch DA, Joseph L (1997). Role of CD44 in nonpalpable T1a and T1b breast cancer. Human Pathology.

[40] Bànkfalvi A, Terpe HJ, Breukelmann D, Bier B, Rempe D, Pschadka G (1998). Gains and losses of CD44 expression during breast carcinogenesis and tumour progression. Histopathology.

[41] Hinz N, Jücker M, [DOI:10.1186/s12964-019-0450-3] (2019). Distinct functions of AKT isoforms in breast cancer: a comprehensive review. Cell Commun Signal.

[42] Boxer RB, Stairs DB, Dugan KD, Notarfrancesco KL, Portocarrero CP, Keister BA (2006). Isoform-specific requirement for Akt1 in the developmental regulation of cellular metabolism during lactation. Cell Metab.

[43] Perou CM, Sørlie T, Eisen MB, van de Rijn M, Jeffrey SS, Rees CA (2000). Molecular portraits of human breast tumours. Nature.

[44] Rädler PD, Wehde BL, Triplett AA, Shrestha H, Shepherd JH, Pfefferle AD (2021). Highly metastatic claudin-low mammary cancers can originate from luminal epithelial cells. Nature Communications.

[45] Lodillinsky C, Infante E, Guichard A, Chaligne R, Fuhrmann L, Cyrta J (2015). p63/MT1-MMP axis is required for in situ to invasive transition in basal-like breast cancer. Oncogene.

